# Does Cell-Type-Specific Silencing of Monoamine Oxidase B Interfere with the Development of Right Ventricle (RV) Hypertrophy or Right Ventricle Failure in Pulmonary Hypertension?

**DOI:** 10.3390/ijms25116212

**Published:** 2024-06-05

**Authors:** Paulin Brosinsky, Jacqueline Heger, Akylbek Sydykov, Astrid Weiss, Stephan Klatt, Laureen Czech, Simone Kraut, Ralph Theo Schermuly, Klaus-Dieter Schlüter, Rainer Schulz

**Affiliations:** 1 Physiologisches Institut, Justus-Liebig-Universität, 35392 Gießen, Germany; jacqueline.heger@physiologie.med.uni-giessen.de (J.H.); laureen.czech@physiologie.med.uni-giessen.de (L.C.); klaus-dieter.schlueter@physiologie.med.uni-giessen.de (K.-D.S.); rainer.schulz@physiologie.med.uni-giessen.de (R.S.); 2 Excellence Cluster Cardiopulmonary System (ECCPS), Justus-Liebig-Universität, 35392 Gießen, Germany; akylbek.sydykov@innere.med.uni-giessen.de (A.S.); astrid.weiss@innere.med.uni-giessen.de (A.W.); simone.kraut@innere.med.uni-giessen.de (S.K.); ralph.schermuly@innere.med.uni-giessen.de (R.T.S.); 3 Vascular Research Centre, Goethe Universität, 60590 Frankfurt, Germany; klatt@vrc.uni-frankfurt.de

**Keywords:** pulmonary hypertension, monoamine oxidase, right heart

## Abstract

Increased mitochondrial reactive oxygen species (ROS) formation is important for the development of right ventricular (RV) hypertrophy (RVH) and failure (RVF) during pulmonary hypertension (PH). ROS molecules are produced in different compartments within the cell, with mitochondria known to produce the strongest ROS signal. Among ROS-forming mitochondrial proteins, outer-mitochondrial-membrane-located monoamine oxidases (MAOs, type A or B) are capable of degrading neurotransmitters, thereby producing large amounts of ROS. In mice, MAO-B is the dominant isoform, which is present in almost all cell types within the heart. We analyzed the effect of an inducible cardiomyocyte-specific knockout of MAO-B (cmMAO-B KO) for the development of RVH and RVF in mice. Right ventricular hypertrophy was induced by pulmonary artery banding (PAB). RV dimensions and function were measured through echocardiography. ROS production (dihydroethidium staining), protein kinase activity (PamStation device), and systemic hemodynamics (in vivo catheterization) were assessed. A significant decrease in ROS formation was measured in cmMAO-B KO mice during PAB compared to Cre-negative littermates, which was associated with reduced activity of protein kinases involved in hypertrophic growth. In contrast to littermates in which the RV was dilated and hypertrophied following PAB, RV dimensions were unaffected in response to PAB in cmMAO-B KO mice, and no decline in RV systolic function otherwise seen in littermates during PAB was measured in cmMAO-B KO mice. In conclusion, cmMAO-B KO mice are protected against RV dilatation, hypertrophy, and dysfunction following RV pressure overload compared to littermates. These results support the hypothesis that cmMAO-B is a key player in causing RV hypertrophy and failure during PH.

## 1. Introduction

Cardiac hypertrophy occurs as a result of a variety of heart diseases, including pulmonary arterial hypertension (PAH). PAH increases right ventricular (RV) afterload, inducing—if prolonged—RV hypertrophy (RVH). With sustained pressure overload, pathological remodeling of the RV occurs, leading to RV dilation and, finally, RV failure (RVF) [1]. While smaller amounts of reactive oxygen species (ROS) act as signaling molecules and contribute to RVH, excessive ROS formation contributes to the transition of adaptive to maladaptive hypertrophy (maladaptive remodeling) and the development of RVF [2,3]. Excessive ROS formation can induce cardiac dysfunction by activating stress protein kinases, through oxidative modification of contractile proteins, by disturbing metabolism and the intracellular ion homeostasis, or by damaging mitochondria and thereby inducing cell death [4].

Apart from a number of cytosolic enzymes, mitochondria also contribute to increased ROS formation [5], and scavenging of mitochondrial ROS attenuates pressure overload- or hypoxia-induced RVH and/or RVF [6]. Mitochondrial ROS are not only derived from the respiratory chain complexes but also from a variety of other mitochondrial proteins. Some of these proposed proteins are uncoupling proteins (UCPs), including p66shc and monoamine oxidases (MAOs) [2,3]. While the importance of UCP, namely UCP2, for ROS formation is debated [3], its importance for the development of RVF during pressure overload has been established [7]. On the contrary, while there is no doubt that p66shc contributes to ROS formation, especially in stress situations [8,9], p66shc has no impact on RVH or RVF during pressure overload [10], which, in part, might be related to its low expression in cardiomyocytes [11].

MAOs are located at the outer mitochondrial membrane where they degrade neurotransmitters to produce ROS. There are two isoforms, MAO-A and MAO-B, presenting 92% of sequence identity [12,13]. MAO-B, which is expressed to a greater extent in the myocardium of mice and humans [14], differs from MAO-A with respect to substrate specificity and inhibitor sensitivity. The two MAO isoforms have common substrates, such as dopamine but also specific substrates. MAO-B can metabolize 1-methyl histamine [15], produced by the histamine-N-methyltransferase [16], while MAO-A metabolizes serotonin (or 5-hydroxytryptamin, 5-HT) and catecholamines (for review, see [17]). MAO requires flavin adenine dinucleotide as a cofactor that is reduced by the reaction and subsequently re-oxidized by oxygen and water, generating hydrogen peroxide [18]. MAO can also form reactive aldehydes, such as 4-hydroxynonenal, as a byproduct of catecholamine metabolism through cardiolipin peroxidation inside mitochondria in primary cardiomyocytes.

While the importance of MAOs in the pathophysiology of left ventricular diseases is well-established [19,20,21,22], data on their importance for RV diseases are rare, but involvement in PH has been proposed [23]. In rats, PH secondary to monocrotaline injection [24], sugen5416/hypoxia, or pulmonary artery banding [25] upregulates MAO-A expression in the pulmonary vasculature and the failing RV. The MAO-A inhibitor clorgyline reduces RV afterload and pulmonary vascular remodeling in sugen/hypoxia rats through reduced pulmonary vascular proliferation and oxidative stress, resulting in improved RV stiffness and relaxation and reversed RV hypertrophy. In rats with PAB, clorgyline has no direct effect on the RV [25].

MAOs are expressed in all cardiac cells, including cardiomyocytes, fibroblasts, and endothelial and vascular smooth muscle cells (for review, see [3]). A permanent cardiomyocyte-specific knockout of MAO-A reduces the incidence of catecholamine-induced arrhythmias in mice [26]. Recently, we demonstrated that a lack of cardiomyocyte-specific MAO-B reduces left ventricular infarct size in vitro [27] and in vivo [28]. Information on the importance of cardiomyocyte-specific MAOs in RV disease is absent. We therefore investigated the effects of MAO-B-mediated ROS formation on RV dilatation and function in response to pulmonary artery banding in the inducible cardiomyocyte-specific MAO-B knockout mouse model [27].

## 2. Results

To determine the role of MAO-B during RVH, Myh6-MCreM_x_MAOB^fl/fl^ mice were used to induce a cardiomyocyte-specific knockout of MAO-B (cmMAO-B KO) and compared to Cre-negative littermates (MAO-B^fl/fl^). Pulmonary artery banding (PAB) was performed to achieve pressure overload, and SHAM-operated animals served as controls.

Body weight and heart rate were similar among the different groups of mice (Table S1). Also, the effect of PAB on left ventricular function was comparable between MAO-B^fl/fl^ and cmMAO-B KO mice (Table S1). Pulmonary artery banding resulted in a similar increase in RV systolic pressure in MAO-B^fl/fl^ and cmMAO-B KO mice measured during RV catheterization (Figure S2).

### 2.1. Effect of cmMAO-B Knockout on Reactive Oxygen Species Formation

**Figure 1:**
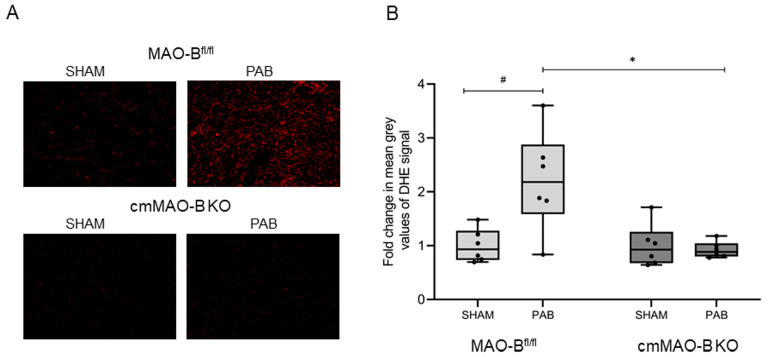
Increased ROS production in response to PAB was abrogated in cmMAO-B KO mice. Representative images (20× microscope’s magnification) of cryosections of RV tissue stained with dihydroethidium (DHE) dye from PAB- or SHAM-operated MAO-B^fl/fl^ and cmMAO-B KO mice (**A**). Quantification of ROS formation in RV tissue from MAO-B^fl/fl^ (SHAM and PAB n = 6 each) and cmMAO-B KO (SHAM and PAB n = 6 each) mice by measuring mean grey values of the DHE signal. Data are presented as mean fold change compared to MAO-B^fl/fl^ SHAM ± SD, * and #: *p* < 0.05 analyzed through two-side ANOVA (**B**).

MAO-B is known to produce ROS during oxidative deamination of neurotransmitters. In certain stress situations, like for left ventricular diseases, the importance of ROS generated by MAOs was already described [22]. To investigate the influence of MAO-B during RV pressure overload, intracellular ROS detection was performed for MAO-B^fl/fl^ and cmMAO-B KO mice. Following three weeks of PAB, ROS formation increased in MAO-B^fl/fl^ mice. In contrast, the effect was completely blunted in cmMAO-B KO mice (Figure 1).

In concordance with the proposed substrates of MAO-B, histamine and ethanolamine concentrations were increased in cmMAO-B KO mice following three weeks of PAB (LC-MS/MS analysis) when compared to MAO-B^fl/fl^ mice (Figure S3).

### 2.2. Effect of cmMAO-B Knockout on Right Ventricular Dimension and Function

RV pressure overload can induce RV remodeling, finally leading to RVF. It was demonstrated that MAO-B is a key regulator for cardiac structural and functional disarrangement in the left ventricle [29]. The role of cmMAO-B for the development of RVH and/or RVF is unknown.

**Figure 2:**
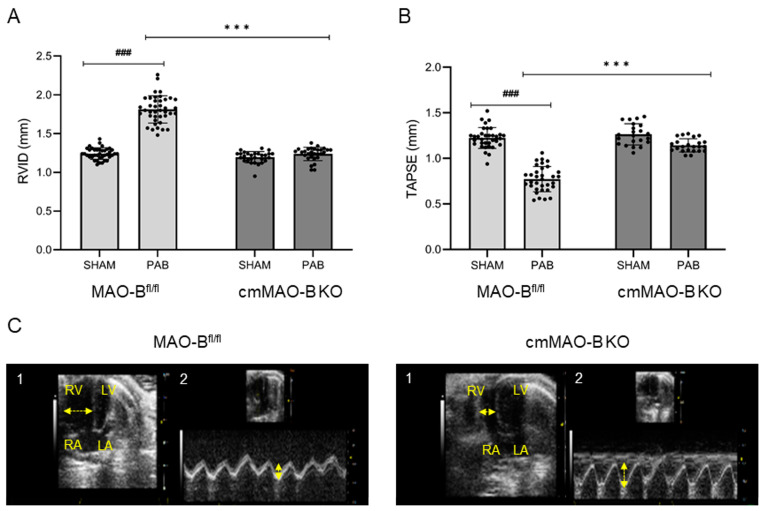
PAB-operated cmMAO-B KO mice were unaffected in their RV dimension and systolic function. RV geometry and function of MAO-B^fl/fl^ (SHAM n = 33, PAB n = 33) and cmMAO-B KO (SHAM n = 22, PAB n = 23) mice were measured through echocardiography three weeks after PAB or SHAM surgery. Data are shown for right ventricular inner diameter (RVID in mm) (**A**) and tricuspid annular plane systolic excursion (TAPSE in mm) (**B**). The dots represent the individual data points. Data represent the mean ± SD, ### and ***: *p* < 0.005 analyzed through two-side ANOVA. Images of apical four-chamber view (1) and the systolic excursion of the tricuspid valve (TAPSE) (2) of one representative MAO-B^fl/fl^ and cmMAO-B KO heart (**C**). LA: left atrium, LV: left ventricle, RA: right atrium, RV: right ventricle. Horizontal arrows represent RVID; vertical arrows represent TAPSE.

Echocardiographic analysis revealed that banded MAO-B^fl/fl^ mice showed a significant increase in RV inner diameter (RVID) and a decrease in RV systolic function (TAPSE), both effects being absent in cmMAO-B KO mice (Figure 2).

**Figure 3:**
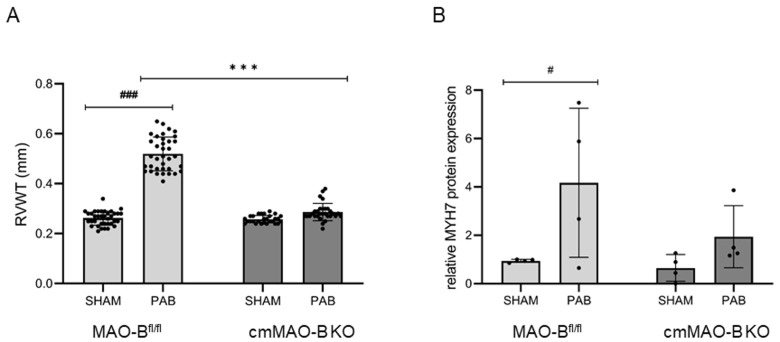
Cardiomyocyte-specific deletion of MAO-B protected the hearts from RVH. Three weeks after SHAM/PAB surgery, mice were evaluated for RVH development. Echocardiography was used to determine right ventricular wall thickness RVWT (in mm). Each dot represents the individual data point. Data represent the mean ± SD for MAO-B^fl/fl^ (SHAM n = 36, PAB n = 36) and cmMAO-B KO mice (SHAM n = 28, PAB n = 30) (**A**). The Jess Simple Western system was used to detect the hypertrophy marker Myosin Heavy Chain (MYH) 7 at the protein level in the right part of the septum of MAO-B^fl/fl^ and cmMAO-B KO mice (n = 4 for each of the four conditions) normalized to vinculin. Data represent the mean ± SD relative to the MAO-B^fl/fl^ SHAM group (**B**). Both diagrams were analyzed through two-side ANOVA, #: *p* < 0.05, ### and ***: *p* < 0.005.

RV wall thickness (RVWT, echocardiography) was increased in MAO-B^fl/fl^ mice; again, the effect was blunted in cmMAO-B KO mice (Figure 3A). The lack of increased cardiomyocyte hypertrophy in cmMAO-B KO mice compared to MAO-B^fl/fl^ mice during PAB was confirmed on the protein level measuring the hypertrophy marker Myosin Heavy Chain (MYH) 7 in the right part of the septum (The Jess Simple Western system) (Figure 3B).

### 2.3. Effect of cmMAO-B Knockout on Protein Kinase Activity during PAB

Because ROS production was increased in MAO-B^fl/fl^ mice during PAB, we assessed the protein kinase activity to identify the de-regulated kinases. Redox-sensitive kinases are known to be involved in cardiac hypertrophy [30,31], so it was assumable that the loss of MAO-B could have an impact. Interestingly, differences in kinase activity could be observed within the types of surgery as well as within the genotypes, as the activities of the kinase were influenced by the cardiomyocyte ablation of MAO-B as well as by PAB (Figure S4). However, in order to assess the role of cmMAO-B during RVH, the focus was mainly on the PAB groups.

**Figure 4:**
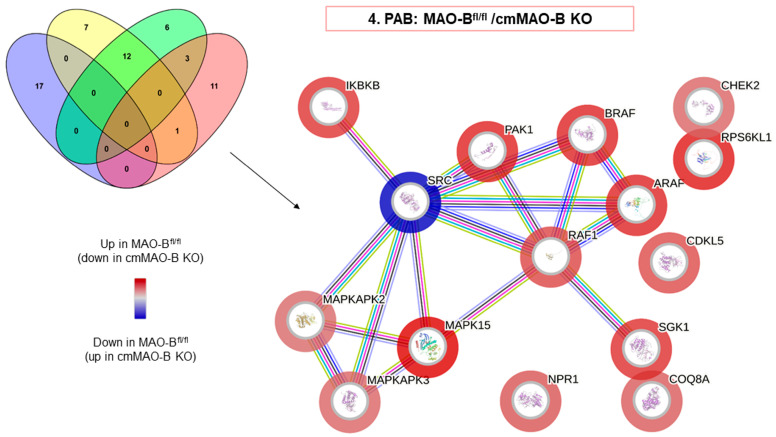
The activity of the kinases involved in cardiac hypertrophy was decreased in cmMAO-B KO mice in response to pressure overload. Functional protein association networks of kinases were analyzed in the right part of the septum of mice due pressure overload. MAO-B^fl/fl^ and cmMAO-B KO mice underwent PAB surgery (n = 4, for each group). After three weeks, protein lysates from the right part of the septum were prepared for kinome profiling. Sequential two-group comparisons were performed to identify kinases, which are differentially regulated because of the genetic background and in response to pressure overload. The resulting hits were uploaded into the String protein database webpage to create images representing functional relationships revealing protein, i.e., kinase, association networks. The Venn diagram highlights the number of kinases that are significantly (*p* < 0.05) de-regulated in not only one but multiple two-group comparisons. For the reactome diagram, the outer color reflects the kinase activity highlighted for PAB: MAO-B^fl/fl^ vs. cmMAO-B KO.

The involvement of ROS as indirect secondary messengers during RVH has already been described [32]. The Ras/Raf/MAPK(MEK)/ERK signaling pathway represents an important signal cascade regulating cardiac hypertrophy [33,34], and half of the de-regulated kinases belong to this MAP kinase signaling pathway (Figure 4, Table S2). In particular, RAF kinases (ARAF/BRAF/RAF1) have already been identified as key regulators during cardiac hypertrophy in mice [30,35].

The activity of protein kinases, which are considered to be involved in cardiac hypertrophy, was increased in MAO-B^fl/fl^ mice during PAB when compared to cmMAO-B KO mice. In addition, all three RAF kinases were significantly downregulated after PAB in cmMAO-B KO mice (Figure 4).

### 2.4. Effect of cmMAO-B Knockout on Cardiomyocyte Function

Next, we examined the contraction behavior of cardiomyoyctes as RV geometry and function were impaired in MAO-B^fl/fl^ mice after PAB.

Isolated RV cardiomyocytes were analyzed from PAB- and SHAM-operated mice. Cardiomyocyte contraction, relaxation, and shortening velocities were not affected by pressure overload, and no significant differences were detected between MAO-B^fl/fl^ and cmMAO-B KO mice (Table 1).

## 3. Discussion

### 3.1. Main Findings

The current study aims to clarify the role of cardiomyocyte MAO-B in the development of RV hypertrophy and failure. The main findings of this study are (1) that RV pressure overload increases myocardial ROS formation in a MAO-B-dependent manner; (2) that functions of isolated RV cardiomyocytes are neither affected by pressure overload nor by MAO-B knockout; and (3) that the development of RV hypertrophy and failure upon pressure overload is abolished by an inducible cardiomyocyte-specific knockout of MAO-B.

### 3.2. MAO and ROS Formation

In isolated mitochondria from mice hearts, we and others could demonstrate both MAO-A and MAO-B expression, with MAO-B being the dominant isoform [3]. However, cardiomyocyte MAO-A also exerts cardiac effects; cardiomyocyte-specific MAO-A inhibition exerts an anti-arrhythmic effect by enhancing diastolic calcium handling under catecholamine stress. Mechanistically, this is facilitated by a reduction in ROS generation, consequently leading to decreased oxidation of type II protein kinase A and calmodulin kinase II [26]. Thus, MAO-A can also contribute to myocardial ROS formation under certain stress conditions (catecholamines). However, with pressure overload as used in the present study, increased myocardial ROS formation seen in MAO-B^fl/fl^ mice was completely abolished following cardiomyocyte-specific knockout of MAO-B. Therefore, the contribution of MAOs to stress-induced ROS formation depends on the underlying stimulus.

### 3.3. MAO Substrates

Mice deficient for both MAO-A and MAO-B demonstrate increased tissue levels of serotonin, norepinephrine, dopamine, and phenylethylamine [36], and genetic ablation of MAO-A increases the serotonin concentration in the blood and tissue in rats [37]. Similarly, blockade of MAO by drugs used for other indications (e.g., antidepressants) can alter histamine levels in mice hearts [38]. Here, we demonstrated that cardiomyocyte-specific knockout of MAO-B increased myocardial histamine and ethanolamine levels, confirming the above substrates for MAO-B and their usage for the formation of myocardial ROS. Interestingly, histamine profoundly impacts the pathophysiology of the heart, resulting in hypertension-induced cardiac hypertrophy (for review, see [39]) through activation of histamine-H2 receptors [40] and histamine-H2 receptor polymorphisms, which altered heart failure development in patients [41]. However, despite the increase in histamine levels following knockout of MAO-B in cardiomyocytes, hypertrophic growth induced by pressure overload following pulmonary artery banding was decreased in cmMAO-B KO mice.

### 3.4. MAO and Pulmonary Hypertension

Data on the importance of MAOs for RV diseases are rare, but involvement in PH has been proposed [23]. In rats, PH secondary to monocrotaline injection [24], sugen5416/hypoxia, or pulmonary artery banding [25] upregulated MAO-A expression in the pulmonary vasculature and the failing RV. The MAO-A inhibitor clorgyline reduced RV afterload and pulmonary vascular remodeling in sugen/hypoxia rats, while clorgyline had no direct effect on the RV following PAB [25]. This is in contrast to the findings of the present study, where inducible cmMAO-B KO preserved RV geometry and function following PAB. Potential explanations for the observed difference could relate to (1) species differences (rats vs. mice), (2) genetic vs. pharmacological approaches, (3) the mode of pressure overload (sugen/hypoxia vs. banding), or (4) as discussed in Section 3.2., substrate availability (catecholamines vs. histamine).

### 3.5. MAO and RV Geometry

In hearts [42] as well as in isolated cardiomyocytes [43] of spontaneously hypertensive rats, MAO activity is significantly increased even before the development of cardiac hypertrophy [43]. Increased MAO activity might represent an early event in the development of cardiac hypertrophy [42] due to its potential impact on cardiac metabolism [44], as cardiac hypertrophy normally goes along with a metabolic switch to preferential use of carbohydrates rather than fatty acids [45,46,47]. In the present study, RV hypertrophy during PAB was dependent on ROS produced by MAO-B, as cardiomyocyte-specific MAO-B knockout abolishes both ROS formation and RV hypertrophy (measured with echocardiography and on the protein level). Data on MAO-dependent effects on myocardial hypertrophy are supported by the measured alterations in protein kinase activity; while PAB increased the activity of redox-sensitive protein kinases involved in hypertrophic growth [31,33], this effect of PAB was blunted in the myocardium from mice with the cardiomyocyte-specific knockout of MAO-B.

For instance, recently, it was shown that BRAF was elevated in humans with heart failure. In addition, cardiomyocyte-specific activation of BRAF in mice led to hypertrophy [30]. Furthermore, a cardiomyocyte-specific KO of BRAF resulted in protection against cardiomyocyte hypertrophy, induced by angiotensin II, in mice [35].

Here, we were able to confirm the connection of activated BRAF and hypertrophy, as these kinases were downregulated in myocardium without cmMAO-B compared to their respective littermates during pressure overload.

In mice, transverse aortic constriction or doxorubicin intoxication led to an increase in left ventricular dimensions (end-diastolic, end-systolic left ventricular diameter) [21,29]. Either global knockout of MAO-B [29] or its pharmacological blockade [21] attenuated the stress-induced changes in left ventricular geometry. Similarly, in the present study, cardiomyocyte-specific knockout of MAO-B abolished the pressure-overload-induced increase in RV diameter.

### 3.6. MAO and Cardiomyocyte Function

It is accepted that a functional impact of hypertrophy is an increased contractility, a common feature of patients with PAH [48] and animals with pressure overload [49]. In a previously published study, we indeed described an improved function of wildtype RV cardiomyocytes following three weeks of PAB [50]. However, whether the observed effect in the above study was ROS-dependent or secondary to changes in cardiac metabolism remains unknown. On the contrary, increased ROS formation can directly reduce cardiomyocyte function through oxidative modification of contractile proteins [51,52]. In the present study, although myocardial ROS were increased with PAB and remained unaffected following cardiomyocyte-specific MAO-B knockout, neither intervention altered the function of isolated RV cardiomyocytes. Thus, the amount of ROS formed by MAO-B during PAB appears to be insufficient to directly modify contractile proteins.

In conclusion, cmMAO-B KO mice were protected against RV dilatation, hypertrophy, and dysfunction following RV pressure overload compared to littermates. These results support the hypothesis that cmMAO-B is a key player in causing RV hypertrophy and failure during PH.

## 4. Materials and Methods

### 4.1. Animals and Ethical Concerns

The conditions of the used animals in the present study conform to the Guide for the Care and Use of Laboratory Animals published by the US National Institutes of Health (NIH publication No. 85-23, revised 1996) and were authorized by the “Regierungspräsidium Gießen” (GI 20/1 Nr. G 31/2019). Mice were bred in the animal facility of the Physiological Institute in Gießen, Germany. Generation, breeding, and induction of knockout mice were performed as previously described in detail [27]. Cardiomyocyte-specific knockout was induced by tamoxifen feeding; Myh6-MCreM_x_MAO-B^fl/fl^ mice (cmMAO-B KO) were fed 400 mg/kg tamoxifen citrate for two weeks, followed by ten weeks of standard chow. Cre-negative MAO-B^fl/fl^ littermates that underwent the same protocol were used as control mice. In the present study, 12–18-week-old female and male mice were used, which were kept in a 12 h light/dark cycle and had free access to standard chow and drinking water, unless otherwise indicated. A schematic overview of the experimental design is shown in Figure S1.

### 4.2. Pulmonary Artery Banding (PAB) In Vivo

The operations were performed as previously described in detail [53]. Thirty minutes prior to the surgery, analgesic buprenorphine hydrochloride (Temgesic^®^, 0.1 mg/kg, Sigma-Aldrich, Steinheim, Germany) was given subcutaneously. To initiate the inhalation anesthesia, 3–4% isoflurane supplemented with 100% oxygen were used and maintained at 1.5–2.5% during the surgery. Before the mice were placed on a heating surface, they were intubated and so mechanically ventilated by using the mouse ventilator MiniVent type 845 (Hugo Sachs Elektronik, March-Hugstetten, Germany). Via the second intercostal space, a left anterolateral thoracotomy was performed. Afterwards, a small titanium clip (Hemoclip^®^, Edward Weck & Co., Inc., Research Triangle Park, NC, USA) was placed with a modified hemoclip applier around the pulmonary trunk. In this way, a 65–70% construction of the pulmonary artery could be achieved. Subsequently, the chest and skin were closed with 6.0 prolene sutures. The SHAM group underwent the same procedure without the application of the hemoclip.

### 4.3. Echocardiography

Three-week-postoperative transthoracic echocardiography was performed by using the Vevo2100 high-resolution imaging system equipped with a 30 MHz transducer (VisualSonics, Toronto, ON, Canada). Inhalation anesthesia was initiated with 3–4% and maintained with 1.5–2% isoflurane in oxygen. The heart rate was monitored by taping all legs to ECG electrodes, and the temperature of the mice was controlled during the procedure. To evaluate the right heart function in vivo, right ventricular wall thickness, right ventricular internal diameter, and tricuspid annular plane systolic excursion were measured. Calculations were performed offline with the software Vevo LAB (version 5.5.0).

### 4.4. Invasive Hemodynamic Measurement

Three weeks after PAB or SHAM operations, hemodynamic measurements were conducted under anesthesia. The animals were anaesthetized with 3–4% isoflurane in oxygen and ventilated with a rodent ventilator (Harvard Apparatus, Holiston, MA, USA). Maintenance of anesthesia could be achieved with 2–3% isoflurane supplemented with oxygen. The mice were laid supine on a heating platform with three legs taped to electrocardiogram electrodes for monitoring of the heart rate. A rectal thermometer (Indus Instruments, Houston, TX, USA) was used to control the body temperature. The heating pad helped to keep the body temperature at 36.5–37.5 °C. Invasive hemodynamic measurements were performed using a micro pressure catheter (Millar instruments, Houston, TX, USA). Systemic arterial pressure was measured via the right carotid artery.

### 4.5. Isolation and Culture of Adult Mouse Ventricular Cardiomyocytes

As described by Bøtker et al., cardiomyocytes were isolated from the control group and the cmMAO-B KO group [54]. Following to the anesthesia with 4–5% isoflurane and the cervical dislocation, hearts were extracted, rapidly rinsed with 4 °C cold 0.9% NaCl, and attached on a cannula of the Langendorff apparatus. With retrograde perfusion in the Langendorff system containing collagenase and calcium-free buffer with a pH of 7.4 at 37 °C (in mmol/L: 10 Glucose monohydrate D^+^, 25 Hepes, 2.5 KCl, 1.2 KH_2_PO_4_, 1.2 MgSO_4_·7H_2_O, 110 NaCl), hearts were digested. Thereafter, the right ventricular wall, left ventricular wall, and ventricular septum were separated. The ventricular tissues were minced and incubated for another 5 min in the recirculating buffer. The septum was stored and frozen at −80 °C. The two suspensions were filtered through a 200 μm nylon mesh and via centrifugation separated from other cells. Within these washing steps, cells were resuspended in culture medium (in mmol/L: 2.5 CaCl_2_-dihydrate, 5 Glucose, 10 Hepes, 4.7 KCl, 1.2 KH_2_PO_4_, 0.8 MgSO_4_, 118 NaCl and 1.9 Na-Pyruvate) by step-wise increasing the concentrations of calcium up to 1 mM. Subsequently, cells were plated on laminin-coated 35 mm culture dishes (Falcon, type 3001). To remove non-attached cardiomyocytes, cells were washed with fresh culture medium after 45 min. The cardiomyocytes were cultured in this medium and ready for analysis; they were kept in an incubator at 37 °C, 5.5% CO_2_, and a humidity of 95% until then.

### 4.6. Determination of Cell Contraction

Cell contraction behavior of MAO-B^fl/fl^ and cmMAO-B KO animals was analyzed at room temperature by using a cell-edge-detection system. The isolated and cultured cardiomyocytes were stimulated via two AgCl electrodes with biphasic electrical stimuli constructed out of two equal but opposite rectangular 50 V stimuli of 0.5 ms duration. With a voltage of 2 Hz each cell was stimulated and measured four times. For the cell shortening calculation, the mean of these measurements was taken. Additionally, cell lengths were analyzed by using a line camera, recorded at 500 Hz. The results were represented as cell shortening and normalized to diastolic cell length (dL/L(%)).

### 4.7. Metabolome Analysis: Sample Extraction

Heart tissues were removed from the mice and immediately frozen in liquid nitrogen. Next, the frozen tissues were homogenized on dry ice and stored at −80 °C. For metabolite extraction, 10–20× the volume of ice-cold methanol extraction buffer was added to the samples (e.g., 20 mg of tissue equals 200–400 µL of extraction buffer). The methanol extraction buffer contains 100% methanol with 1 mM of TCEP, 1 mM of ascorbic acid, 0.1% formic acid, and 0.1 µM of internal standards. The samples were vortexed and sonicated on ice until completely homogenous. Next, the samples were put on ice for 15 min and subsequently centrifuged at 14,000 rpm for 10 min at 4 °C to remove protein. The supernatant was transferred to another tube. Two times the volume of MilliQ-water was added. The samples were frozen at −80 °C, followed by a sublimation step on a freeze-dryer using the Alpha 3-4 LSCbasic system (Martin Christ, Osterode am Harz, Germany). Next, the dried samples were reconstituted in 80 µL of 200 mM of boric acid buffer, shortly vortexed, and incubated on ice for 15 min. Next, 20 µL of AQC reagent was added (from AccQ-Taq Derivatization Kit, Milford, CT, USA, Waters application note). The samples were shortly vortexed and derivatized for 10 min at 55 °C. After the incubation step, the samples were put back on ice and centrifuged again at 14,000 rpm for 10 min at 4 °C. At last, the supernatant was transferred to MS glass vials.

### 4.8. LC-MS/MS Analysis

Metabolites were identified and quantified by using a 1290 Infinity II Bio LC system coupled to a 6495C QQQ MS in dynamic MRM mode (both Agilent Technologies, Waldbronn, Germany). In detail, metabolites were identified with authentic standards and/or via retention time, elution order from the column, and 1–2 transitions. LC separation of metabolites was performed on an Agilent Zorbax Extend RR HD 1.8 µm (2.1 × 150 mm) column with a solvent system of 0.1% formic acid in water (A) and 0.1% formic acid in acetonitrile (B). The LC gradient was 19 min long, with the following schedule: 0 min 1% B, 2 min 1% B, 9 min 15% B, 14 min 30% B, 16 min 60% B, and 17 min 65% B until 19 min 1% B. The flow-rate was set to 300 µL/min, and the column temperature was set to 30 °C. Data acquisition was performed in positive ionization mode. The gas temperature was set to 290 °C, and the gas flow was set to 20 L/min. The nebulizer was set to 45 psi. The sheath gas flow was set to 11 L/min, with a temperature of 400 °C. The capillary voltage was set to 3800 V with a nozzle voltage of 500 V. The voltages of the High-Pressure RF and Low-Pressure RF were set to 150/60 V, respectively.

### 4.9. Detection of Intracellular ROS

To detect intracellular ROS, cell-permeable fluorescent dye dihydroethidium (DHE) was used. Cryosections of RV from MAO-B^fl/fl^ and cmMAO-B KO were incubated with DHE (dissolved in 1× PBS) for 5 min at 37 °C in the dark. Subsequently, the cryosections were washed with PBS and fixed with Dako Fluorescent Mounting Medium (Dako, North America, Inc., Carpinteria, CA, USA). Slides were then placed under a fluorescence microscope (BZ-X810 Keyence, Neu-Isenburg, Germany) and analyzed with the Quantity One 1-D Analysis Software (BIO-RAD, Version 4.6.6).

### 4.10. Peptide-Based Kinase Activity Assay

Protein isolation and peptide-based kinase activity assays for tyrosine as well as serine/threonine kinases (using the PamStation device, Pamgene, ’s Hertogenbosch, The Netherlands) were conducted according to the manufacturer’s instructions, as previously described [55,56,57]. As the right ventricle consists of a small amount of tissue and this tissue had already been used for other analyses, the right parts of the septum were used for the subsequently performed analysis of kinase activity and protein expression. This meant that the same set of mice could be used. It was assumed that tissue from the right ventricle was mainly included on the right side of the septum. For lysis of the right part of the septum, the tissues were mechanically homogenized using the Precellys tissue homogenizers and 1.4 mm ceramic beads (zirconium oxide) at 5500 rpm for 10 s two times, in the presence of 100 µL of M-PER lysis buffer (Thermo Fisher Scientific, Waltham, MA, USA) supplemented with protease and phosphatase inhibitor cocktail (Thermo Fisher Scientific). Afterwards, the homogenates were incubated for 1 h at 4 °C and centrifuged at 13,000 rpm at 4 °C for 15 min, and the supernatant was immediately aliquoted, flash-frozen, and stored at −80 °C until the time point of measurement. The protein concentration was determined using a bicinchoninic acid (BCA) protein assay kit (Thermo Fisher Scientific) according to the manufacturer’s instructions. Then, 10 µg of protein lysate was applied on the tyrosine kinase PamChips (2 µg for serine/threonine kinase, respectively) to investigate the kinase-mediated phosphorylation of the substrate peptides. The assay was performed according to the manufacturer’s instructions using the Evolve software (PamGene, Evolve 3, version 3.1.0.5). Based on the pattern of phosphorylation for the four experimental conditions, bioinformatic analyses through BioNavigator software (PamGene, BioNavigator 6, version 6.3.67.0) using protein databases allowed for a prediction of kinases upstream of these events.

### 4.11. Jess Simple Western System (ProteinSimple, San Jose, CA, USA)

This is an automated, capillary-based size separation immunoassay. This technology was used to detect the hypertrophy marker Myosin Heavy Chain 7 (MYH7) (Invitrogen, Waltham, MA, USA, NOQ7.5.4D, diluted 1:50) at the protein level in the right part of the septum of mice. MYH7 protein was normalized to Vinculin (Sigma-Aldrich, V9131, diluted 1:125,000). The standard reagent pack containing the components for preparing the samples and the size marker (12–230 kDa) was applied according to the manufacturer’s instructions. For each capillary, 2.4 µL of the sample with an average concentration of 5.6 µg/µL (resulting in a total protein amount of 13.4 µg on average) was mixed with 0.6 µL of 5× fluorescence master mix (provided as part of the standard reagent pack). Next, the samples were denatured for 5 min at 95 °C, quickly centrifuged, and stored on ice. The plate was loaded with all components according to the manufacturer’s pipetting scheme and afterwards centrifuged for 5 min at 1000× *g*. The plate as well as the cartridge (i.e., capillaries) were placed in the Jess device (ProteinSimple, San Jose, CA, USA), and a fully automated separation together with the immunodetection of proteins took place within three hours. Detection was based on chemiluminescence using horseradish peroxidase-coupled secondary antibodies. The light emission was detected using a CCD camera and then analyzed using the Compass software (version 4.1.0, Protein Simple). The results, i.e., protein expression reflected by band intensities, are displayed in traditional lane view as well as electropherograms, which allow for quantification by defining the respective area under the curve.

### 4.12. Statistical Analysis

Data presented in the figures are expressed in means ± SD and individual data points. Data presented in the tables are expressed as means ± SD. A *p*-value < 0.05 is considered to indicate a significant difference. All data were analyzed through two-side ANOVA. The program GraphPad Prism 9.4.1 was used for statistical analysis.

## Figures and Tables

**Table 1:** cmMAO-B KO and pressure overload had no influence on contraction behavior of cardiomyocytes. Cell shortening of isolated cardiomyocytes from RV three weeks after PAB (MAO-B^fl/fl^ n = 6, cmMAO-B KO n = 5) or SHAM (MAO-B^fl/fl^ n = 6, cmMAO-B KO n = 6) surgery (n = number of mice, 36 cardiomyocytes were analyzed per animal). Data of diastolic cell length (Ldiast in µm), contraction velocity (Con Vel in µm/s), relaxation velocity (Rel Vel in µm/s), and load-free cell shortening (quantified as percent shortening amplitude normalized to the diastolic cell length of individual cells (dL/L(%)) are shown. Data represent the mean ± SD.

	RV	Ldiast (µm)	Con Vel (µm/s)	Rel Vel (µm/s)	dL/L (%)
MAO-B^fl/fl^	SHAM	109.93 ± 9.79	202.63 ± 51.36	166.05 ± 45.05	8.53 ± 1.82
	PAB	111.32 ± 7.23	182.99 ± 46.88	151.03 ± 45.33	7.65 ± 7.65
cmMAO-B KO	SHAM	112.08 ± 11.66	215.58 ± 72.75	180.14 ± 59.07	9.10 ± 2.44
	PAB	108.36 ± 11.00	183.64 ± 48.88	152.43 ± 45.10	8.30 ± 2.08

## Data Availability

The data presented in this study are available on request from the corresponding author.

## Supplementary Materials

The following supporting information can be downloaded at: https://www.mdpi.com/article/10.3390/ijms25116212/s1.
