# Supplementary material for: Does Cell-Type-Specific Silencing of Monoamine Oxidase B Interfere with the Development of Right Ventricle (RV) Hypertrophy or Right Ventricle Failure in Pulmonary Hypertension?
Source: Int J Mol Sci. 2024 Jun 5;25(11):6212. doi: 10.3390/ijms25116212 (PMC11172614; doi:10.3390/ijms25116212)
Supplement: Supplementary file 1 [file ijms-25-06212-s001.zip › ijms-2991638-supplementary.pdf]

Supplement

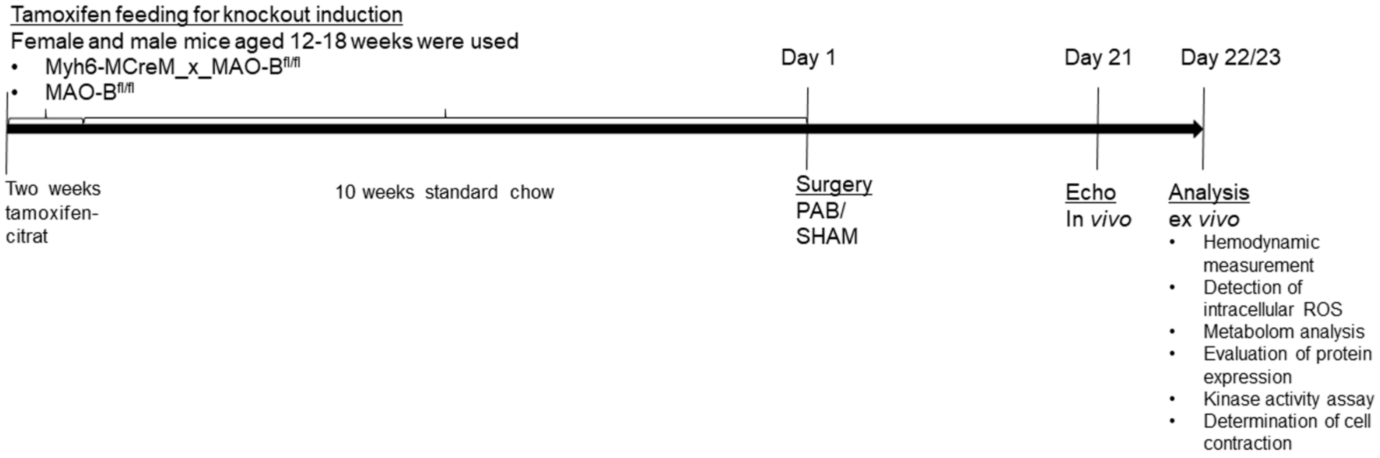

Figure S1: Schematic overview of the experimental design. For the tamoxifen-inducible, cardiomyocyte-specific knockout of MAO-B female and male mice were used with an age of 12-18 weeks. After the 12-week protocol of knockout-induction, mice of both genotypes underwent either PAB or SHAM surgery. Three weeks after the surgery, animals (of all four conditions) were echocardiographically examined (echo) and used for further analysis in the following two days.

Table S1: Pressure overload or cmMAO-B KO had no effect on body weight, heart rate or global cardiac function. Echocardiographic measurements of MAO-B$^{fl/fl}$ (SHAM n= 33 and PAB n= 31) and cmMAO-B KO (SHAM n= 22 and PAB n= 25) mice three weeks after SHAM/PAB surgery. Data are given for BW (body weight in g), VTI (velocity time integral), HR (heart rate in beats per min), CO (cardiac output in ml/min) and CI (cardiac index in ml/min/g). Data are means ± SD, #: p< 0.05; ###: p< 0.005 between SHAM and PAB in MAO-B$^{fl/fl}$. $: p< 0.05; $$$: p< 0.005 between SHAM and PAB in cmMAO-B KO. Statistics were analyzed by two-side ANOVA.

		BW (g)	VTI (mm/s)	HR	CO (ml/min)	CI (ml/min/g)
MAO-B$^{fl/fl}$	*SHAM*	26.62 ± 4.09	32.26 ± 5.33	487 ± 53.36	19.74 ± 4.76	0.74 ± 0.15
	PAB	25.91 ± 3.40	27.04 ± 4.36###	497 ± 42.46	16.5 ± 3.06###	0.65 ± 0.15#
cmMAO-B KO	*SHAM*	26.61 ± 3.98	34.33 ± 5.86	503 ± 62.78	21.98 ± 6.00	0.83 ± 0.19
	PAB	25.92 ± 3.75	29.69 ± 5.53$$$	493 ± 44.00	18.46 ± 5.65$	0.69 ± 0.20$

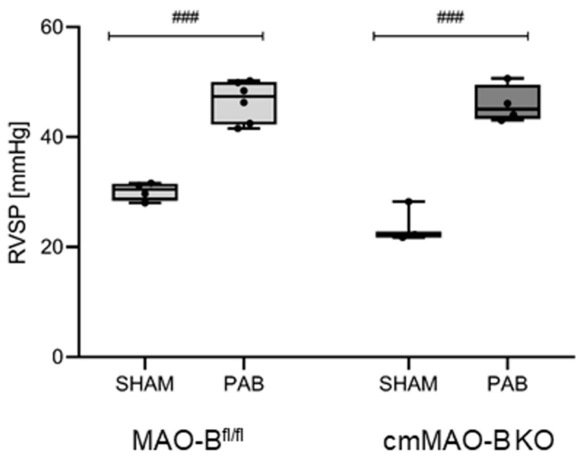

Figure S2: Pressure overload was similarly induced in MAO-B$^{fl/fl}$ and cmMAO-B KO mice. Systemic hemodynamics in mice exposed either to SHAM (MAO-B$^{fl/fl}$ n= 4, cmMAO-B KO n= 3) or PAB (MAO-B$^{fl/fl}$ n= 5, cmMAO-B KO n= 4) operation for three weeks. Data are given for right ventricular systolic pressure (RVSP in mmHg). Data represent the mean ± SD, ### p< 0.005 analyzed by two-side ANOVA.

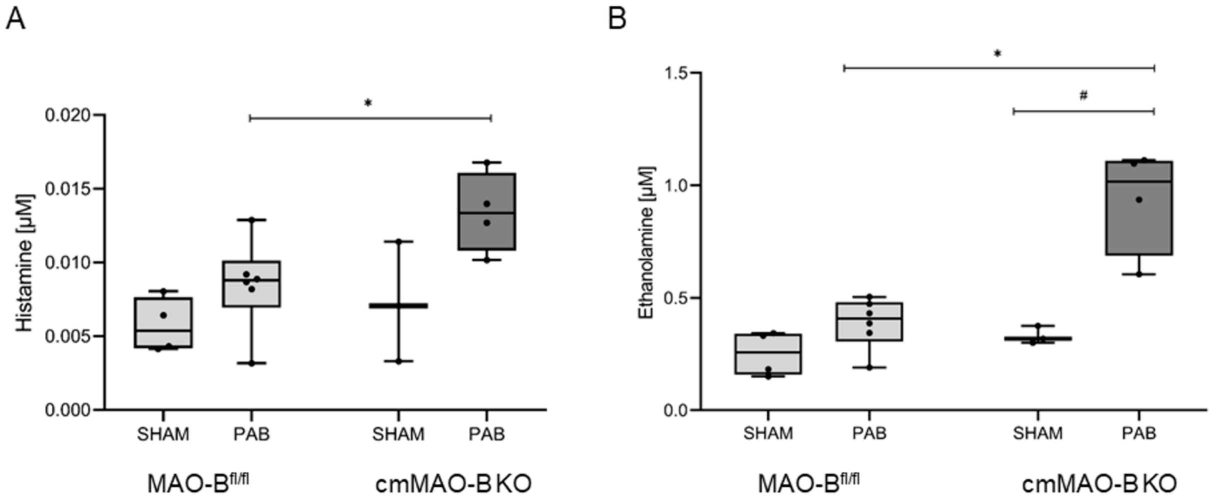

Figure S3 cmMAO-B used histamine and ethanolamine as substrates especially during PAB. LC-MS/MS analysis to identify and quantify metabolites from RV tissue. Concentrations (in µM) of MAO-B substrates histamine (A) and ethanolamine (B) were analyzed three weeks postoperative. Data are given for MAO-B$^{fl/fl}$ (SHAM n= 4, PAB n= 6) and cmMAO-B KO (SHAM n= 3, PAB n= 4) mice. Data represent the mean ± SD, * and #: p <0.05 analyzed by two-side ANOVA.

Table S2 Term description for PAB: MAO-B$^{fl/fl}$/cmMAO-B KO. Functional protein association networks of kinases were analyzed in the right part of the septum of mice due to pressure overload. MAO-B$^{fl/fl}$ and cmMAO-B KO mice underwent PAB surgery (n = 4, for each group). After three weeks, protein lysates from the right part of the septum were prepared for kinome profiling. Sequential two-group comparisons were performed to identify kinases, which are differentially regulated because of the genetic background and in response to pressure overload. The resulting hits were uploaded into the String protein database webpage to create images representing functional relationships revealing protein, i.e., kinase, association networks. The top 20 term descriptions associated with the respective kinases of the network are listed.

p38MAPK events
Signaling by NTRK1 (TRKA)
Signalling to ERKs
RAF activation
MAP2K and MAPK activation
Signaling by moderate kinase activity BRAF mutants
Signaling by high-kinase activity BRAF mutants
Signaling by BRAF and RAF1 fusions
Paradoxical activation of RAF signaling by kinase inactive BRAF
Signaling downstream of RAS mutants
Signaling by RAF1 mutants
SHOC2 M1731 mutant abolishes MRAS complex function
Gain-of-function MRAS complexes activate RAF signaling
VEGFA-VEGFR2 Pathway
Signaling by Receptor Tyrosine Kinases
C-type lectin receptors (CLRs)
MAPK family signaling cascades
Negative regulation of MAPK pathway
MAP kinase activation
Negative feedback regulation of MAPK pathway

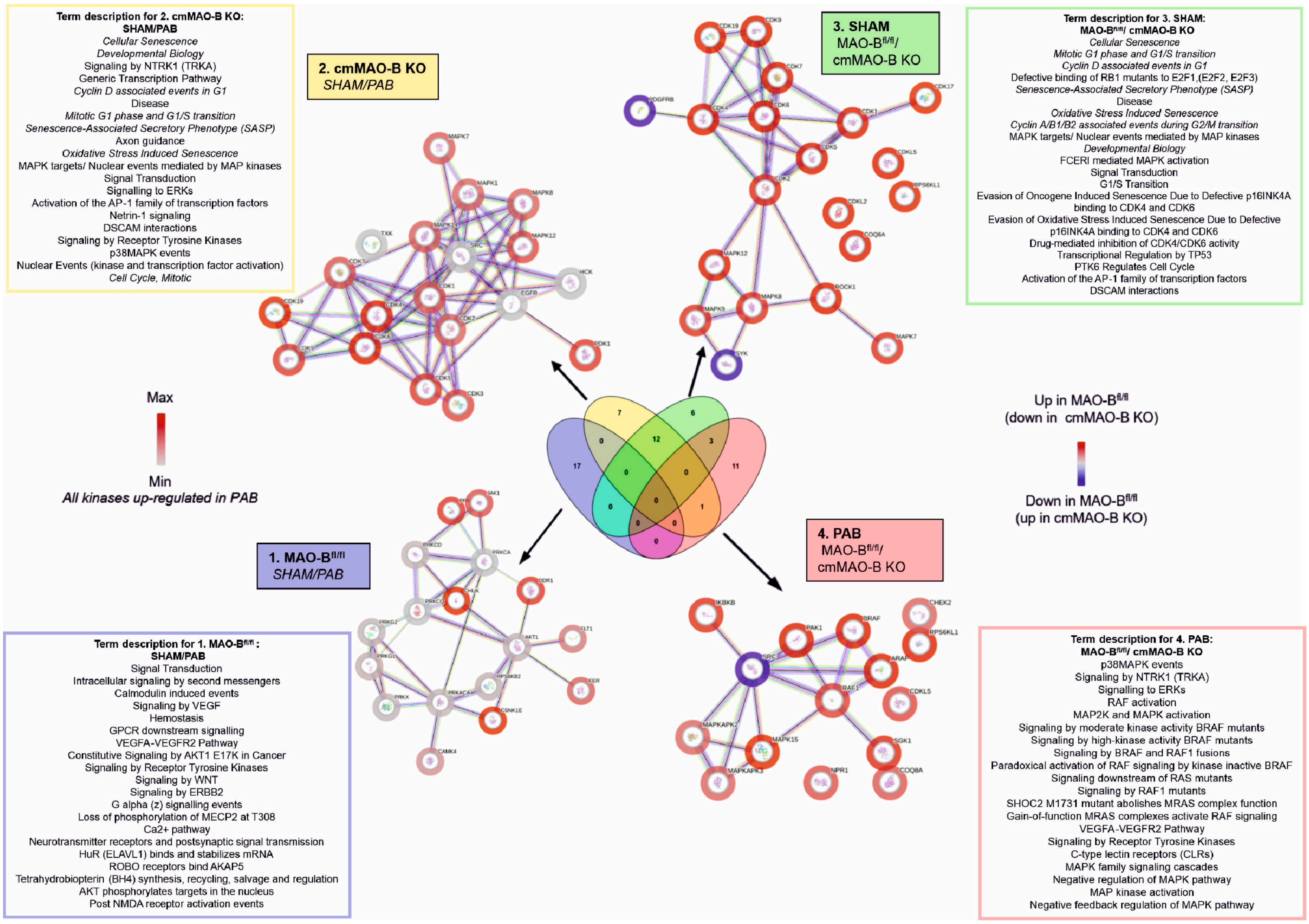

Term description for 2. cmMAO-B KO:
SHAM/PAB
Cellular Senescence
Developmental Biology
Signaling by NTRK1 (TRKA)
Generic Transcription Pathway
Cyclin D associated events in G1
Disease
Mitotic G1 phase and G1/S transition
Senescence-Associated Secretory Phenotype (SASP)
Axon guidance
Oxidative Stress Induced Senescence
MAPK targets/ Nuclear events mediated by MAP kinases
Signal Transduction
Signalling to ERKs
Activation of the AP-1 family of transcription factors
Netrin-1 signaling
DSCAM interactions
Signaling by Receptor Tyrosine Kinases
p38MAPK events
Nuclear Events (kinase and transcription factor activation)
Cell Cycle, Mitotic
2. cmMAO-B KO
SHAM/PAB
3. SHAM
MAO-B^fl/fl/
cmMAO-B KO
Term description for 3. SHAM:
MAO-B^fl/fl/ cmMAO-B KO
Cellular Senescence
Mitotic G1 phase and G1/S transition
Cyclin D associated events in G1
Defective binding of RB1 mutants to E2F1,(E2F2, E2F3)
Senescence-Associated Secretory Phenotype (SASP)
Disease
Oxidative Stress Induced Senescence
Cyclin A/B1/B2 associated events during G2/M transition
MAPK targets/ Nuclear events mediated by MAP kinases
Developmental Biology
FCERI mediated MAPK activation
Signal Transduction
G1/S Transition
Evasion of Oncogene Induced Senescence Due to Defective p16INK4A binding to CDK4 and CDK6
Evasion of Oxidative Stress Induced Senescence Due to Defective p16INK4A binding to CDK4 and CDK6
Drug-mediated inhibition of CDK4/CDK6 activity
Transcriptional Regulation by TP53
PTK6 Regulates Cell Cycle
Activation of the AP-1 family of transcription factors
DSCAM interactions
Max
Min
All kinases up-regulated in PAB
Up in MAO-B^fl/fl
(down in cmMAO-B KO)
Down in MAO-B^fl/fl
(up in cmMAO-B KO)
1. MAO-B^fl/fl
SHAM/PAB
4. PAB
MAO-B^fl/fl/
cmMAO-B KO
Term description for 1. MAO-B^fl/fl:
SHAM/PAB
Signal Transduction
Intracellular signaling by second messengers
Calmodulin induced events
Signaling by VEGF
Hemostasis
GPCR downstream signalling
VEGFA-VEGFR2 Pathway
Constitutive Signaling by AKT1 E17K in Cancer
Signaling by Receptor Tyrosine Kinases
Signaling by WNT
Signaling by ERBB2
G alpha (z) signalling events
Loss of phosphorylation of MECP2 at T308
Ca2+ pathway
Neurotransmitter receptors and postsynaptic signal transmission
HuR (ELAVL1) binds and stabilizes mRNA
ROBO receptors bind AKAP5
Tetrahydrobiopterin (BH4) synthesis, recycling, salvage and regulation
AKT phosphorylates targets in the nucleus
Post NMDA receptor activation events
Term description for 4. PAB:
MAO-B^fl/fl/ cmMAO-B KO
p38MAPK events
Signaling by NTRK1 (TRKA)
Signalling to ERKs
RAF activation
MAP2K and MAPK activation
Signaling by moderate kinase activity BRAF mutants
Signaling by high-kinase activity BRAF mutants
Signaling by BRAF and RAF1 fusions
Paradoxical activation of RAF signaling by kinase inactive BRAF
Signaling downstream of RAS mutants
Signaling by RAF1 mutants
SHOC2 M1731 mutant abolishes MRAS complex function
Gain-of-function MRAS complexes activate RAF signaling
VEGFA-VEGFR2 Pathway
Signaling by Receptor Tyrosine Kinases
C-type lectin receptors (CLRs)
MAPK family signaling cascades
Negative regulation of MAPK pathway
MAP kinase activation
Negative feedback regulation of MAPK pathway

Figure S4: cmMAO-B KO and PAB had an impact on kinase activities**.** Functional protein association networks of kinases were carried out in the right part of the septum of mice due to MAO-B knockout and/or PAB surgery. MAO-B$^{fl/fl}$ and cmMAO-B KO mice underwent either SHAM or PAB surgery (n= 4, each of the four groups). After three weeks protein lysates from the right part of the septum were prepared for kinome profiling. Sequential two-group comparisons (indicated by 1. to 4.) were performed to identify kinases which are differentially regulated either because of the genetic background, i.e. MAO-B$^{fl/fl}$ versus cmMAO-B KO and/or by the type of surgery, i.e. SHAM versus PAB. The resulting hits were uploaded into the String protein database webpage to create images representing functional relationships revealing protein, i.e. kinase, association networks. Here, the outer color reflects the kinase activity: Highest activity shown in dark red, lowest activity in blue. The following groups were analyzed: 1. MAO-B$^{fl/fl}$: SHAM/PAB, 2. cmMAO-B KO: SHAM/PAB, 3. SHAM: MAO-B$^{fl/fl}$/cmMAO-B KO and 4. PAB: MAO-B$^{fl/fl}$/cmMAO-B KO.

String database webpage: https://string-db.org/, please cite PMID: 30476243

Biovenn webpage: https://bioinfogp.cnb.csic.es/tools/venny/index.html, please cite *Oliveros, J.C. (2007-2015) Venny. An interactive tool for comparing lists with Venn's diagrams.*
